# (*E*)-1-Diphenyl­methyl­idene-2-[(1*H*-indol-3-yl)methyl­idene]hydrazine

**DOI:** 10.1107/S1600536810020702

**Published:** 2010-06-05

**Authors:** R. Archana, R. Anbazhagan, K. R. Sankaran, A. Thiruvalluvar, R. J. Butcher

**Affiliations:** a PG Research Department of Physics, Rajah Serfoji Government College (Autonomous), Thanjavur 613 005, Tamil Nadu, India; b Department of Chemistry, Annamalai University, Annamalai Nagar 608 002, Tamil Nadu, India; c Department of Chemistry, Howard University, 525 College Street NW, Washington, DC 20059, USA

## Abstract

In the title compound, C_22_H_17_N_3_, the 1*H*-indole unit is essentially planar, with a dihedral angle of 0.95 (10)° between the pyrrole ring and the fused benzene ring. The dihedral angle between the two phenyl rings is 65.09 (10)°. In the crystal, an inter­molecular N—H⋯N hydrogen bond forms an infinite chain in the *b*-axis direction.

## Related literature

For the synthesis, see: Fleming & Harley-Mason (1961 ▶). For the crystal structures of some aromatic azines, for example, acetophenone azine, see: Glaser *et al.* (1995 ▶). For other heterocyclic aldehyde azines, see: Lin *et al.* (2001 ▶). For the crystal structure of symmetrical 1*H*-Indole-3-carbaldehyde azine, see: Rizal *et al.* (2008 ▶).
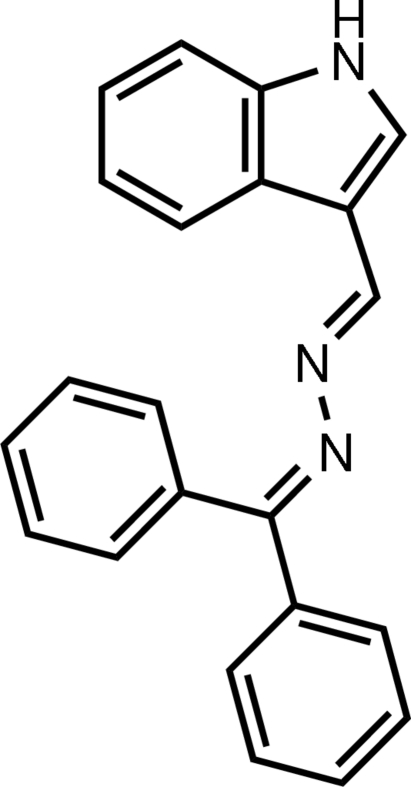


## Experimental

### 

#### Crystal data

C_22_H_17_N_3_
*M* *_r_* = 323.39
Orthorhombic, 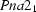

*a* = 24.1594 (3) Å
*b* = 13.8501 (2) Å
*c* = 5.2173 (1) Å
*V* = 1745.76 (5) Å^3^
*Z* = 4
Cu *K*α radiation
μ = 0.58 mm^−1^
*T* = 295 K
0.46 × 0.21 × 0.18 mm

#### Data collection

Oxford Diffraction Xcalibur Ruby Gemini diffractometer
Absorption correction: multi-scan (*CrysAlis PRO*; Oxford Diffraction, 2009 ▶) *T*_min_ = 0.796, *T*_max_ = 1.000
8042 measured reflections
2059 independent reflections
1954 reflections with *I* > 2σ(*I*)
*R*_int_ = 0.015

#### Refinement

*R*[*F* ^2^ > 2σ(*F* ^2^)] = 0.032
*wR*(*F* ^2^) = 0.092
*S* = 1.06
2059 reflections
230 parameters
1 restraint
H atoms treated by a mixture of independent and constrained refinement
Δρ_max_ = 0.12 e Å^−3^
Δρ_min_ = −0.17 e Å^−3^

### 

Data collection: *CrysAlis PRO* (Oxford Diffraction, 2009 ▶); cell refinement: *CrysAlis PRO*; data reduction: *CrysAlis PRO*; program(s) used to solve structure: *SHELXS97* (Sheldrick, 2008 ▶); program(s) used to refine structure: *SHELXL97* (Sheldrick, 2008 ▶); molecular graphics: *ORTEP-3* (Farrugia, 1997 ▶); software used to prepare material for publication: *PLATON* (Spek, 2009 ▶).

## Supplementary Material

Crystal structure: contains datablocks global, I. DOI: 10.1107/S1600536810020702/nk2037sup1.cif

Structure factors: contains datablocks I. DOI: 10.1107/S1600536810020702/nk2037Isup2.hkl

Additional supplementary materials:  crystallographic information; 3D view; checkCIF report

## Figures and Tables

**Table 1:** Hydrogen-bond geometry (Å, °)

*D*—H⋯*A*	*D*—H	H⋯*A*	*D*⋯*A*	*D*—H⋯*A*
N1—H1⋯N2^i^	0.88 (3)	2.18 (2)	3.0069 (19)	159 (3)

Symmetry code: (i) 
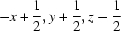
.

**Fig. 1.**
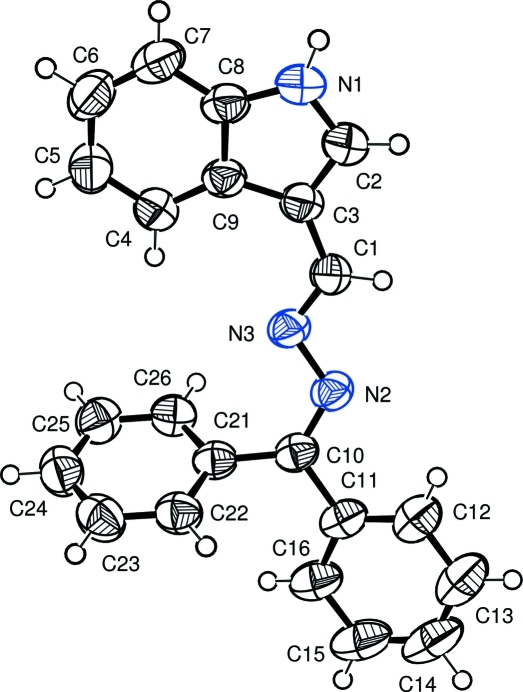
The molecular structure of the title compound, showing the atom-numbering scheme and displacement ellipsoids drawn at the 50% probability level. H atoms are shown as small spheres of arbitrary radius.

**Fig. 2.**
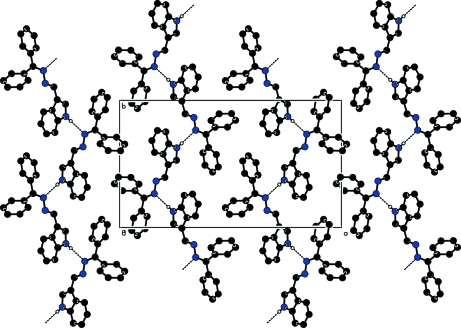
The packing of the title compound, viewed down the c axis. Dashed lines indicate hydrogen bonds. H atoms not involved in hydrogen bonding have been omitted.

## supplementary crystallographic information

### Comment

The title compound is an unsymmetrical indole azine derived from benzophenone, indole-3-carboxaldehyde and hydrazine. Glaser *et al.*, (1995) have reported the crystal structures of some aromatic azines. Lin *et al.*, (2001) have reported heterocyclic aldehyde azines. Rizal *et al.*, (2008) have reported the crystal structure of symmetrical 1*H*-Indole-3-carbaldehyde azine. Herein, we report the crystal structure of the title compound.

In the title molecule (Scheme I, Fig. 1), C_22_H_17_N_3_, the 1*H*-indole unit is almost planar, as the pyrrole ring makes a dihedral angle of 0.95 (10)° with the fused benzene ring. The r.m.s. deviation of a mean plane fitted through all non hydrogen atoms of the indole unit is  0.0096 Å; C3 deviates from this plane by 0.015 (1) Å. The dihedral angle between the two phenyl rings of the diphenylmethylene residue is 65.09 (10)°. The crystal structure is stabilized by intermolecular N1—H1···N2(1/2 - *x*, 1/2 + *y*, -1/2 + *z*) hydrogen bond forming an infinite one-dimensional chain in the b-axis direction (Fig. 2).

### Experimental

The compound was prepared in accord with literature precedents Fleming & Harley-Mason (1961). The mixture of benzophenone hydrazone (1.96 g, 0.01 mol) and indole-3-carboxaldehyde (2.55 g, 0.01 mol) in ethanol was refluxed for 2 h. The mixture was cooled to room temperature over night. The solid obtained was separated, dried and then recrystallized from absolute ethanol. The yield of isolated product was (1.76 g, 79%).

### Refinement

Owing to the absence of any anamalous scatterers in the molecule,  Friedel pairs were merged. The absolute structure in the model was chosen arbitrarily. The N-bound H1 atom was located in a difference Fourier map, and was freely refined. Remaining H atoms were positioned geometrically and allowed to ride on their parent atoms, with C—H = 0.93 Å. *U*_iso_(H) = 1.2*U*_eq_(C).

### Crystal data

**Table:**

C_22_H_17_N_3_	*D*_x_ = 1.230 Mg m^−^^3^
*M**_r_* = 323.39	Melting point: 423 K
Orthorhombic, *P**n**a*2_1_	Cu *K*α radiation, λ = 1.54184 Å
Hall symbol: P 2c -2n	Cell parameters from 6053 reflections
*a* = 24.1594 (3) Å	θ = 4.9–77.4°
*b* = 13.8501 (2) Å	µ = 0.57 mm^−^^1^
*c* = 5.2173 (1) Å	*T* = 295 K
*V* = 1745.76 (5) Å^3^	Needle, pale yellow
*Z* = 4	0.46 × 0.21 × 0.18 mm
*F*(000) = 680	

### Data collection

**Table:**

Oxford Diffraction Xcalibur Ruby Gemini diffractometer	2059 independent reflections
Radiation source: Enhance (Cu) X-ray Source	1954 reflections with *I* > 2σ(*I*)
graphite	*R*_int_ = 0.015
Detector resolution: 10.5081 pixels mm^-1^	θ_max_ = 77.6°, θ_min_ = 4.9°
ω scans	*h* = −30→27
Absorption correction: multi-scan (*CrysAlis PRO*; Oxford Diffraction, 2009)	*k* = −17→17
*T*_min_ = 0.796, *T*_max_ = 1.000	*l* = −5→6
8042 measured reflections	

### Refinement

**Table:**

Refinement on *F*^2^	Primary atom site location: structure-invariant direct methods
Least-squares matrix: full	Secondary atom site location: difference Fourier map
*R*[*F*^2^ > 2σ(*F*^2^)] = 0.032	Hydrogen site location: inferred from neighbouring sites
*wR*(*F*^2^) = 0.092	H atoms treated by a mixture of independent and constrained refinement
*S* = 1.06	*w* = 1/[σ^2^(*F*_o_^2^) + (0.0712*P*)^2^ + 0.0189*P*] where *P* = (*F*_o_^2^ + 2*F*_c_^2^)/3
2059 reflections	(Δ/σ)_max_ = 0.001
230 parameters	Δρ_max_ = 0.12 e Å^−^^3^
1 restraint	Δρ_min_ = −0.17 e Å^−^^3^

### Special details

**Table:**

Geometry. Bond distances, angles *etc*. have been calculated using the rounded fractional coordinates. All su's are estimated from the variances of the (full) variance-covariance matrix. The cell e.s.d.'s are taken into account in the estimation of distances, angles and torsion angles
Refinement. Refinement of *F*^2^ against ALL reflections. The weighted *R*-factor *wR* and goodness of fit *S* are based on *F*^2^, conventional *R*-factors *R* are based on *F*, with *F* set to zero for negative *F*^2^. The threshold expression of *F*^2^ > 2σ(*F*^2^) is used only for calculating *R*-factors(gt) *etc*. and is not relevant to the choice of reflections for refinement. *R*-factors based on *F*^2^ are statistically about twice as large as those based on *F*, and *R*- factors based on ALL data will be even larger.

### Fractional atomic coordinates and isotropic or equivalent isotropic displacement parameters (Å^2^)

**Table:**

	*x*	*y*	*z*	*U*_iso_*/*U*_eq_	
N1	0.25795 (6)	0.62655 (10)	0.3007 (4)	0.0689 (4)	
N2	0.15647 (5)	0.26575 (8)	0.6043 (3)	0.0551 (3)	
N3	0.16478 (5)	0.36551 (8)	0.6361 (3)	0.0550 (4)	
C1	0.20416 (6)	0.39405 (10)	0.4934 (4)	0.0582 (4)	
C2	0.25644 (7)	0.52898 (11)	0.2923 (4)	0.0692 (5)	
C3	0.21904 (6)	0.49427 (10)	0.4681 (4)	0.0578 (4)	
C4	0.15783 (6)	0.59044 (12)	0.7935 (4)	0.0636 (5)	
C5	0.14574 (8)	0.68387 (14)	0.8692 (5)	0.0778 (6)	
C6	0.17085 (8)	0.76319 (13)	0.7519 (5)	0.0794 (7)	
C7	0.20847 (8)	0.75255 (11)	0.5582 (5)	0.0711 (6)	
C8	0.22152 (6)	0.65856 (11)	0.4834 (4)	0.0587 (4)	
C9	0.19622 (5)	0.57731 (10)	0.5974 (3)	0.0540 (4)	
C10	0.11010 (6)	0.23275 (9)	0.6904 (3)	0.0510 (3)	
C11	0.09878 (6)	0.12935 (10)	0.6271 (3)	0.0576 (4)	
C12	0.12405 (8)	0.08728 (13)	0.4155 (4)	0.0715 (6)	
C13	0.11502 (10)	−0.00994 (14)	0.3603 (5)	0.0890 (8)	
C14	0.08040 (10)	−0.06415 (13)	0.5133 (6)	0.0945 (9)	
C15	0.05508 (10)	−0.02335 (13)	0.7208 (6)	0.0891 (8)	
C16	0.06379 (8)	0.07414 (11)	0.7795 (5)	0.0710 (5)	
C21	0.06955 (6)	0.28877 (10)	0.8444 (3)	0.0513 (4)	
C22	0.01336 (6)	0.28961 (11)	0.7848 (4)	0.0609 (4)	
C23	−0.02317 (7)	0.34404 (14)	0.9306 (5)	0.0728 (6)	
C24	−0.00457 (8)	0.39643 (14)	1.1386 (4)	0.0735 (6)	
C25	0.05106 (8)	0.39457 (13)	1.2010 (4)	0.0686 (5)	
C26	0.08756 (6)	0.34155 (11)	1.0559 (3)	0.0593 (4)	
H1	0.2797 (10)	0.6616 (17)	0.204 (6)	0.089 (7)*	
H1A	0.22419	0.34832	0.40160	0.0698*	
H2	0.27765	0.49087	0.18322	0.0830*	
H4	0.14080	0.53787	0.87152	0.0764*	
H5	0.12039	0.69401	1.00059	0.0933*	
H6	0.16173	0.82502	0.80720	0.0953*	
H7	0.22468	0.80575	0.47948	0.0853*	
H12	0.14699	0.12400	0.31071	0.0858*	
H13	0.13237	−0.03826	0.22001	0.1067*	
H14	0.07425	−0.12885	0.47504	0.1133*	
H15	0.03194	−0.06049	0.82360	0.1069*	
H16	0.04622	0.10185	0.92001	0.0852*	
H22	0.00029	0.25361	0.64707	0.0731*	
H23	−0.06054	0.34525	0.88781	0.0873*	
H24	−0.02924	0.43266	1.23595	0.0882*	
H25	0.06382	0.42930	1.34165	0.0823*	
H26	0.12488	0.34091	1.09955	0.0711*	

### Atomic displacement parameters (Å^2^)

**Table:**

	*U*^11^	*U*^22^	*U*^33^	*U*^12^	*U*^13^	*U*^23^
N1	0.0668 (7)	0.0567 (7)	0.0833 (9)	−0.0144 (6)	0.0116 (8)	0.0021 (8)
N2	0.0562 (6)	0.0416 (5)	0.0675 (7)	0.0010 (4)	−0.0022 (6)	0.0012 (6)
N3	0.0535 (5)	0.0425 (5)	0.0689 (8)	−0.0018 (4)	0.0006 (5)	0.0014 (6)
C1	0.0501 (6)	0.0493 (6)	0.0751 (10)	0.0016 (5)	0.0050 (7)	−0.0012 (7)
C2	0.0642 (8)	0.0578 (8)	0.0856 (11)	−0.0070 (6)	0.0161 (9)	−0.0013 (9)
C3	0.0483 (6)	0.0514 (7)	0.0737 (9)	−0.0034 (5)	0.0032 (7)	0.0006 (7)
C4	0.0556 (7)	0.0622 (8)	0.0731 (10)	−0.0023 (6)	0.0038 (8)	−0.0011 (8)
C5	0.0673 (9)	0.0766 (10)	0.0894 (14)	0.0047 (8)	0.0079 (9)	−0.0178 (10)
C6	0.0784 (10)	0.0573 (8)	0.1025 (16)	0.0033 (7)	−0.0065 (11)	−0.0201 (10)
C7	0.0747 (9)	0.0509 (7)	0.0877 (12)	−0.0090 (7)	−0.0107 (10)	−0.0036 (8)
C8	0.0535 (6)	0.0529 (7)	0.0697 (9)	−0.0084 (5)	−0.0072 (7)	0.0002 (7)
C9	0.0445 (6)	0.0507 (6)	0.0668 (9)	−0.0037 (5)	−0.0064 (6)	−0.0003 (7)
C10	0.0535 (6)	0.0432 (5)	0.0562 (7)	−0.0007 (5)	−0.0090 (6)	0.0058 (6)
C11	0.0620 (7)	0.0449 (6)	0.0660 (8)	−0.0027 (5)	−0.0177 (7)	0.0019 (7)
C12	0.0800 (10)	0.0593 (8)	0.0752 (11)	0.0023 (7)	−0.0156 (9)	−0.0079 (8)
C13	0.1040 (14)	0.0647 (10)	0.0982 (16)	0.0089 (9)	−0.0288 (13)	−0.0240 (11)
C14	0.1079 (15)	0.0465 (8)	0.129 (2)	−0.0049 (9)	−0.0440 (16)	−0.0108 (11)
C15	0.1001 (14)	0.0516 (9)	0.1155 (19)	−0.0200 (9)	−0.0224 (13)	0.0108 (11)
C16	0.0786 (10)	0.0512 (7)	0.0833 (11)	−0.0120 (7)	−0.0120 (10)	0.0077 (8)
C21	0.0551 (7)	0.0453 (6)	0.0535 (7)	−0.0041 (5)	−0.0037 (6)	0.0089 (5)
C22	0.0557 (7)	0.0612 (7)	0.0658 (8)	−0.0039 (6)	−0.0070 (7)	0.0032 (8)
C23	0.0559 (7)	0.0787 (10)	0.0837 (12)	0.0040 (7)	−0.0001 (9)	0.0088 (10)
C24	0.0738 (10)	0.0731 (9)	0.0737 (11)	0.0080 (8)	0.0134 (9)	0.0029 (9)
C25	0.0808 (10)	0.0693 (9)	0.0557 (8)	−0.0044 (8)	0.0030 (8)	−0.0016 (8)
C26	0.0596 (7)	0.0627 (8)	0.0556 (7)	−0.0049 (6)	−0.0055 (6)	0.0036 (7)

### Geometric parameters (Å, °)

**Table:**

N1—C2	1.353 (2)	C21—C26	1.393 (2)
N1—C8	1.371 (3)	C21—C22	1.393 (2)
N2—N3	1.4060 (16)	C22—C23	1.388 (3)
N2—C10	1.2906 (19)	C23—C24	1.381 (3)
N3—C1	1.271 (2)	C24—C25	1.383 (3)
N1—H1	0.88 (3)	C25—C26	1.375 (2)
C1—C3	1.440 (2)	C1—H1A	0.9300
C2—C3	1.374 (3)	C2—H2	0.9300
C3—C9	1.443 (2)	C4—H4	0.9300
C4—C5	1.384 (3)	C5—H5	0.9300
C4—C9	1.393 (2)	C6—H6	0.9300
C5—C6	1.396 (3)	C7—H7	0.9300
C6—C7	1.367 (3)	C12—H12	0.9300
C7—C8	1.395 (2)	C13—H13	0.9300
C8—C9	1.412 (2)	C14—H14	0.9300
C10—C21	1.486 (2)	C15—H15	0.9300
C10—C11	1.4949 (19)	C16—H16	0.9300
C11—C12	1.390 (3)	C22—H22	0.9300
C11—C16	1.390 (3)	C23—H23	0.9300
C12—C13	1.394 (3)	C24—H24	0.9300
C13—C14	1.379 (3)	C25—H25	0.9300
C14—C15	1.366 (4)	C26—H26	0.9300
C15—C16	1.400 (2)		
			
N1···N2^i^	3.0069 (19)	C12···H2^ii^	3.0600
N2···N1^ii^	3.0069 (19)	C13···H2^ii^	3.0900
N3···C4	3.226 (2)	C14···H6^v^	2.9300
N3···C26	2.896 (2)	C16···H22	3.0000
N2···H26^iii^	2.9300	C21···H16	2.6800
N2···H1^ii^	2.18 (2)	C22···H16	2.8100
N2···H26	2.8900	H1···N2^i^	2.18 (2)
N2···H12	2.5000	H1···C10^i^	2.84 (2)
N3···H4	2.7500	H1···C11^i^	3.00 (2)
N3···H26	2.6300	H1···C12^i^	2.96 (3)
C2···C12^i^	3.585 (3)	H1A···C6^viii^	2.9000
C4···N3	3.226 (2)	H1A···C7^viii^	2.7600
C6···C14^iv^	3.470 (3)	H1A···H7^viii^	2.5900
C12···C2^ii^	3.585 (3)	H2···C12^i^	3.0600
C13···C16^iii^	3.474 (4)	H2···C13^i^	3.0900
C14···C6^v^	3.470 (3)	H4···N3	2.7500
C16···C22	3.224 (2)	H5···H23^ix^	2.5400
C16···C13^vi^	3.474 (4)	H6···C14^iv^	2.9300
C22···C25^iii^	3.496 (3)	H7···H1A^vii^	2.5900
C22···C16	3.224 (2)	H12···N2	2.5000
C25···C22^vi^	3.496 (3)	H16···C21	2.6800
C26···N3	2.896 (2)	H16···C22	2.8100
C1···H26^iii^	2.9000	H22···C11	2.9400
C6···H1A^vii^	2.9000	H22···C16	3.0000
C7···H1A^vii^	2.7600	H23···H5^x^	2.5400
C10···H1^ii^	2.84 (2)	H26···N2	2.8900
C11···H22	2.9400	H26···N2^vi^	2.9300
C11···H1^ii^	3.00 (2)	H26···N3	2.6300
C12···H1^ii^	2.96 (3)	H26···C1^vi^	2.9000
			
C2—N1—C8	109.17 (15)	C23—C24—C25	119.44 (18)
N3—N2—C10	115.52 (12)	C24—C25—C26	120.24 (18)
N2—N3—C1	110.08 (13)	C21—C26—C25	121.07 (15)
C2—N1—H1	123.5 (16)	N3—C1—H1A	119.00
C8—N1—H1	127.4 (17)	C3—C1—H1A	119.00
N3—C1—C3	122.71 (15)	N1—C2—H2	125.00
N1—C2—C3	110.22 (16)	C3—C2—H2	125.00
C1—C3—C2	124.23 (16)	C5—C4—H4	121.00
C2—C3—C9	106.52 (13)	C9—C4—H4	121.00
C1—C3—C9	129.06 (15)	C4—C5—H5	119.00
C5—C4—C9	118.18 (16)	C6—C5—H5	119.00
C4—C5—C6	121.3 (2)	C5—C6—H6	119.00
C5—C6—C7	121.87 (18)	C7—C6—H6	119.00
C6—C7—C8	117.23 (17)	C6—C7—H7	121.00
C7—C8—C9	121.87 (17)	C8—C7—H7	121.00
N1—C8—C7	129.89 (17)	C11—C12—H12	120.00
N1—C8—C9	108.24 (13)	C13—C12—H12	120.00
C4—C9—C8	119.58 (14)	C12—C13—H13	120.00
C3—C9—C4	134.58 (14)	C14—C13—H13	120.00
C3—C9—C8	105.84 (13)	C13—C14—H14	120.00
N2—C10—C11	114.91 (13)	C15—C14—H14	120.00
N2—C10—C21	125.15 (12)	C14—C15—H15	120.00
C11—C10—C21	119.94 (12)	C16—C15—H15	120.00
C10—C11—C12	119.79 (14)	C11—C16—H16	120.00
C12—C11—C16	119.41 (15)	C15—C16—H16	120.00
C10—C11—C16	120.80 (15)	C21—C22—H22	120.00
C11—C12—C13	120.02 (18)	C23—C22—H22	120.00
C12—C13—C14	120.1 (2)	C22—C23—H23	120.00
C13—C14—C15	120.36 (19)	C24—C23—H23	120.00
C14—C15—C16	120.3 (2)	C23—C24—H24	120.00
C11—C16—C15	119.8 (2)	C25—C24—H24	120.00
C10—C21—C22	121.76 (14)	C24—C25—H25	120.00
C22—C21—C26	118.48 (14)	C26—C25—H25	120.00
C10—C21—C26	119.76 (13)	C21—C26—H26	119.00
C21—C22—C23	120.14 (17)	C25—C26—H26	119.00
C22—C23—C24	120.62 (17)		
			
C8—N1—C2—C3	0.1 (2)	C7—C8—C9—C4	−1.2 (3)
C2—N1—C8—C7	−179.2 (2)	N2—C10—C11—C12	−24.1 (2)
C2—N1—C8—C9	0.6 (2)	N2—C10—C11—C16	155.04 (17)
C10—N2—N3—C1	−164.25 (15)	C21—C10—C11—C12	156.63 (16)
N3—N2—C10—C11	172.86 (13)	C21—C10—C11—C16	−24.3 (2)
N3—N2—C10—C21	−7.9 (2)	N2—C10—C21—C22	131.69 (18)
N2—N3—C1—C3	174.46 (16)	N2—C10—C21—C26	−48.9 (2)
N3—C1—C3—C2	−171.37 (18)	C11—C10—C21—C22	−49.1 (2)
N3—C1—C3—C9	3.0 (3)	C11—C10—C21—C26	130.34 (15)
N1—C2—C3—C1	174.67 (17)	C10—C11—C12—C13	177.96 (18)
N1—C2—C3—C9	−0.8 (2)	C16—C11—C12—C13	−1.2 (3)
C1—C3—C9—C4	6.0 (3)	C10—C11—C16—C15	−178.12 (19)
C1—C3—C9—C8	−174.06 (18)	C12—C11—C16—C15	1.0 (3)
C2—C3—C9—C4	−178.90 (18)	C11—C12—C13—C14	0.9 (3)
C2—C3—C9—C8	1.10 (19)	C12—C13—C14—C15	−0.5 (4)
C9—C4—C5—C6	0.5 (3)	C13—C14—C15—C16	0.4 (4)
C5—C4—C9—C3	−179.81 (19)	C14—C15—C16—C11	−0.6 (4)
C5—C4—C9—C8	0.2 (2)	C10—C21—C22—C23	−178.96 (16)
C4—C5—C6—C7	−0.2 (4)	C26—C21—C22—C23	1.6 (2)
C5—C6—C7—C8	−0.8 (3)	C10—C21—C26—C25	179.57 (15)
C6—C7—C8—N1	−178.7 (2)	C22—C21—C26—C25	−1.0 (2)
C6—C7—C8—C9	1.5 (3)	C21—C22—C23—C24	−1.2 (3)
N1—C8—C9—C3	−1.04 (19)	C22—C23—C24—C25	0.2 (3)
N1—C8—C9—C4	178.96 (15)	C23—C24—C25—C26	0.5 (3)
C7—C8—C9—C3	178.80 (18)	C24—C25—C26—C21	−0.1 (3)

Symmetry codes: (i) −*x*+1/2, *y*+1/2, *z*−1/2; (ii) −*x*+1/2, *y*−1/2, *z*+1/2; (iii) *x*, *y*, *z*−1; (iv) *x*, *y*+1, *z*; (v) *x*, *y*−1, *z*; (vi) *x*, *y*, *z*+1; (vii) −*x*+1/2, *y*+1/2, *z*+1/2; (viii) −*x*+1/2, *y*−1/2, *z*−1/2; (ix) −*x*, −*y*+1, *z*+1/2; (x) −*x*, −*y*+1, *z*−1/2.

### Hydrogen-bond geometry (Å, °)

**Table:**

*D*—H···*A*	*D*—H	H···*A*	*D*···*A*	*D*—H···*A*
N1—H1···N2^i^	0.88 (3)	2.18 (2)	3.0069 (19)	159 (3)

Symmetry codes: (i) −*x*+1/2, *y*+1/2, *z*−1/2.
